# OTME-22. Bioinformatic evaluation of ECM molecules and angiogenic associate genes in diffuse midline glioma (DMG): mapping the tumour microenvironment

**DOI:** 10.1093/noajnl/vdab070.073

**Published:** 2021-07-05

**Authors:** Nikita Kozhushko, Malwina Jedrysik, Helen Fillmore

**Affiliations:** University of Portsmouth, Portsmouth, UK

## Abstract

Paediatric Diffuse Intrinsic Pontine Glioma (DIPG) is a devastating cancer of an extremely aggressive nature, located in the pontine area of the brain. DIPG primarily affects children, with the average age of diagnosis between 6 and 7 years. Unfortunately, the outlook and overall survival remains bleak. While there has been impressive progress in identifying genes that are central to and drive DIPG growth; there remains several gaps in documenting the DIPG microenvironment landscape. The focus of this study is to begin to examine mRNA expression of genes associated with blood vessel development, angiogenesis, and extracellular matrix molecules (ECM) in normal brain development and DIPG by utilizing publicly available genomic datasets. In-depth bioinformatics from GSE26576 dataset included differential expression and gene ontology (GO) with KEGG pathway analyses using Gene Expression Omnibus (GEO) and DAVID, which have revealed a number of significantly upregulated genes that may affect DIPG angiogenic processes (p<0.05). 38 of such genes from 9 different GO terms were then included in a protein-to-protein interaction network that revealed a surprising connection between *MMP16, CSPG4* and *COL11A1*. Subsequently, using R2 genomic visualisation platform from publicly available single cell RNAseq data we showcased the difference in their individual expression based on the molecular subtypes of DIPG histone 3 (H3) mutation (K27M, wild type and G34R) with a strong statistical significance (p<0.05). Interestingly, during normal paediatric development such genes showed consistent expression, suggesting their potential complications in DIPG angiogenesis. Overall, this bioinformatic approach has led to the identification of a set of interacting genes that will inform our *in vitro* and *in vivo* studies. This information will add to the documentation of the host/tumour microenvironment landscape and our plan is to continue to explore this area to map the spatial and temporal expression of these genes.

